# How to deal with coronavirus disease 2019: A comprehensive narrative review about oral involvement of the disease

**DOI:** 10.1002/cre2.332

**Published:** 2020-10-05

**Authors:** Giorgia Capocasale, Riccardo Nocini, Paolo Faccioni, Dario Donadello, Dario Bertossi, Massimo Albanese, Francesca Zotti

**Affiliations:** ^1^ Section of Dentistry and Maxillofacial Surgery, Department of Surgical Sciences, Paediatrics and Gynaecology University of Verona Verona Italy; ^2^ Section of Ears, Nose and Throat (ENT), Department of Surgical Sciences, Dentistry, Gynecology and Pediatrics University of Verona Verona Italy

**Keywords:** COVID‐19, dysgeusia, oral blister, oral disease

## Abstract

**Objectives:**

The aim of this narrative review was to collect all findings from literature about oral signs and symptoms of COVID‐19, in order to draw a picture of oral involvement of this challenging viral infection, to help oral professionals in a better triage and early diagnosis.

**Material and methods:**

The search for international literature was made including articles written in English and reporting about oral manifestations in patients with a diagnosis of COVID‐19. The publication time was limited to 2019 and 2020, up to May 20, 2020. A narrative review was performed.

**Results:**

Twenty‐three articles were included in this review. Three different oral manifestations were found: taste alteration, oral blister and ulcers, and oral lesions associated with Kawasaki‐like diseases (erythema, bleeding of lips, “strawberry tongue”). The higher expression of Angiotensin‐converting enzyme 2 in the oral cavity and in endothelial cells might be responsible for oral manifestation and the major report of signs and symptoms in the occidental countries.

**Conclusions:**

Detecting oral signs and symptoms of COVID‐19 could be useful to perform a better preliminary triage in dental setting, and in recognizing possible early manifestations of the disease. However, considering the outbreak of COVID‐19 and the consequent difficulty of undergoing oral examinations, the oral manifestations might be misdiagnosed; then, we would encourage oral professionals to perform other studies about this topic.

## 1 INTRODUCTION

The coronavirus disease 2019 (COVID‐19), caused by severe acute respiratory syndrome coronavirus 2 (SARS‐CoV‐2), started from Wuhan, China, in December 2019. On January 30, 2020, the World Health Organization (WHO) announced “a public health emergency of international concern” (Sohrabi et al., 2020) and on March 11, declared the pandemic condition.

This disease had high virulence with the human‐to‐human transmission, with an incubation period ranged from 2 to 14 days (Jiang et al., 2020).

Several studies reported that novel Coronavirus 2019 uses Angiotensin‐converting enzyme 2 (ACE2) as its host receptor (Wan, Shang, Graham, Baric, & Li, 2020; Zhang, Penninger, Li, Zhong, & Slutsky, 2020), used for host cell entry and then, viral replication.

Lung appears to be the target organ and common clinical features of this virus infection were fever, respiratory involvement, dry cough and diarrhea (Jiang et al., 2020). However, the exponential diffusion of the COVID‐19 infection around the world showed some new clinical signs of the disease (e.g., cutaneous [Tang et al., 2020], gastrointestinal [Lee, Huo, & Huang, 2020] manifestations). It was reported that atypical manifestations could be in some cases the first and/or the only manifestations of this disease (Daruich, Martin, & Bremond‐Gignac, 2020; Wang, Wang, Chen, Tao, & Zeng, 2020); therefore, in this context, all specialists can contribute to early diagnosis, then to control outbreaks, identifying early signs and symptoms of COVID‐19 to speed‐up self‐isolation procedures.

Rapid detection of COVID‐19 is crucial to control outbreaks: only by means of an early identification of manifestations and consequent early diagnosis, it is possible an effective and rapid isolation of cases and the appropriate contact tracing.

Indeed, the literature reported that case isolation was more effective when the delay from symptom onset to isolation was short (Hellewell et al., 2020).

The aim of this brief narrative review was to outline the oral signs and symptoms observed in patients diagnosed with COVID‐19 reported in the literature, in order and to better profile the role of oral professionals and what they may have to know. Other studies and reports about this topic are surely required.

## 2 METHODS

Two of the authors (CG, NR) independently performed a literature search on PUBMED and PMC. At the beginning, the following search strategy was uploaded, combining free words and MESH terms: (oral manifestation) AND (COVID19 OR Coronavirus Disease 2019 OR SARS‐CoV‐2). Therewith, in this literature research, studies describing cutaneous manifestation were included, because these are often associated with oral manifestations, as described for some disease (Capocasale et al., 2017; Zotti et al., 2020), and therefore reported by dermatologists.

Considering the timing of the spread of the disease, the publication time was limited to 2019 and 2020, up to May 20, 2020. We had included all types of studies regarding the oral manifestations observed in patients diagnosed with COVID‐19, only literature reviews were excluded. Other exclusion criteria were: articles for which full text was not available, articles were not in English or were grey literature.

Duplicate articles were eliminated and the first evaluation was performed reading only the title and abstract of the studies. Later, all studies considered eligible were included for full‐text evaluation and only studies considered eligible by both authors were included in the review.

From those retrieved during the search, additional references were identified by a manual search among the cited references.

## 3 RESULTS

From the first search resulted in 515 works, 508 resulted after checking of duplicates. By searching in references resulted, 17 were retrieved. Finally, according to inclusion and exclusion criteria, 23 were included for the narrative review. The literature selection process was reported in the diagram (Table 1).

**TABLE 1:** Flow chart of literature search

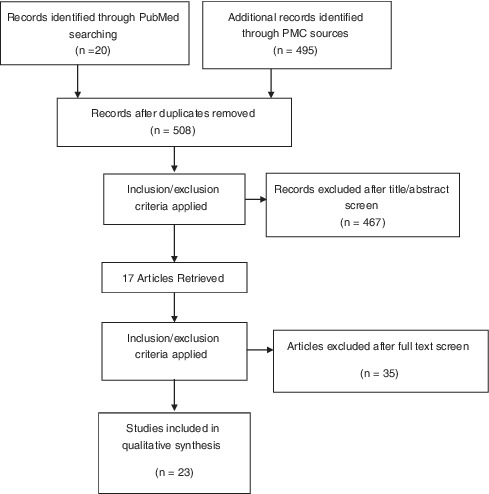

Results were synthesized in Table 2: name of the first author and year, country of the diagnosed cases, oral sign or symptom reported, sample size, patients' age, gender and studies design were inserted. In addition, crucial information found in these studies were also presented. Given that the studies were published between December 2019 and May 20, 2020, results were set in the table in alphabetical order, by the surnames of the first authors, and divided considering the oral signs or symptoms described.

**TABLE 2:** Synthesis of results

Author, year	Oral manifestation	Country	N. oral manifestation/sample size	Gender (M‐F)	Mean age (years)	Study design/other crucial information
Beltrán‐Corbellini et al., 2020	Taste alteration	Spain	28/79	48 M‐31 F	61.6	Case control/self‐reported
Bénézit et al., 2020	Taste alteration	France	63/68	NR	NR	Cross‐sectional study/self‐reported
Gelardi, Trecca, Cassano, & Ciprandi, 2020	Taste alteration	Italy	52/72	39 M‐33 F	49.7	Case series/medical history
Giacomelli et al., 2020	Taste alteration	Italy	17/59	40 M‐19 F	60	Cross‐sectional study/self‐reported
Hjelmesæth & Skaare, 2020	Taste alteration	Norway	3/4	NR	NR	Case series/self‐reported. Family cases
Jang et al., 2020	Taste alteration	Korea	1/1	M/42	42	Case report/self‐reported
Lechien et al., 2020	Taste alteration	Europe	342/417	154 M‐263 F	36.9 ± 11.4	Cross‐sectional study/National Health and Nutrition Examination Survey
Lee, Huo, & Huang, 2020	Taste alteration	Korea	488/3.191	1.161 M‐2.030 F	44	Retrospective study/self‐reported
Mao et al., 2020	Taste alteration	China	12/214	87 M‐127 F	52.7	Retrospective study/medical history
Melley, Bress, & Polan, 2020	Taste alteration	USA	1/1	F	59	Case report/medical history‐self‐reported
Paderno et al., 2020	Taste alteration	Italy	284/508	285 M‐223 F	55.15	Cross‐sectional study/self‐reported
Roland, Gurrola, Loftus, Cheung, & Chang, 2020	Taste alteration	USA	96/145	40 M‐105 F	39	Cross‐sectional study/self‐reported survey
Vaira et al., 2020	Taste alteration	Italy	62/320	NR	NR	Cross‐sectional study/medical history
Vaira, Salzano, Deiana, & De Riu, 2020	Taste alteration	Italy	39/72	27 M‐45 F	49.2	Cross‐sectional study/taste test
Vaira et al., 2020	Taste alteration	Italy	17/33	11 M‐22 F	51.8	Cross‐sectional study/self‐administrate taste test
Villalba et al., 2020	Taste alteration	France	1/2	1 M‐1 F	82.5	Case series/medical history
Wee et al., 2020	Taste alteration	Singapore	35/154	NR	NR	Cross‐sectional study/self‐reported
Yan, Faraji, Prajapati, Ostrander, & DeConde, 2020	Taste alteration	USA	40/59	NR	NR	Cross‐sectional study/self‐reported
Martín Carreras‐Presas, Amaro Sánchez, López‐Sánchez, Jané‐Salas, & Somacarrera Pérez, 2020	Oral blister and ulcers	Spain	1/3	1 F	65	Case series/EM‐like lesion
Hedou et al., 2020	Oral blister and ulcers	France	1/103	32 M‐71 F	47	Cross‐sectional study/HSV in intubated patient
Jimenez‐Cauhe et al., 2020	Oral blister and ulcers	Spain	3/4	4 F	66.65	Case series/EM‐like lesion
Rivera‐Figueroa, Santos, Simpson, & Garg, 2020	Kawasaki‐like disease	USA	1/1	M	5	Case report/incomplete KD
Verdoni et al., 2020	Kawasaki‐like disease	Italy	5/10	7 M‐3 F	7.5	Retrospective study/4 patients with a classic form and 1 with incomplete had oral manifestation

Abbreviations: EM‐like lesion, Erythema Multiforme‐like eruption; F, female; HSV, Herpes simplex virus; KD, Kawasaki disease; M, male; NR, not reported.

In the sections below, oral manifestations observed were described.

### 3.1 Taste alteration

A pool of 5,399 patients diagnosed with COVID‐19 resulted from studies selected, from those 1,581 subjects (29.28%) reported taste alteration as a symptom.

Taste alteration was found to be the most reported and thus described oral manifestation during the COVID‐19 with a range of prevalence between 5.6% (Mao et al., 2020) and 92.64% (Bénézit et al., 2020). Studies analyzed outlined a varied picture of taste alterations, such as ageusia, hypogeusia and dysgeusia. Also, Jang et al. (2020) brought to the attention of scientific community a case of a 42‐year‐old male who reported both loss of taste and smell and but also having a metallic taste. He was only this manifestation of the disease, and he screened for COVID‐19 only because he reported to had proximity with a COVID‐19 positive subject.

Paderno et al. (2020) reported that in 11% of cases patients mentioned the taste alteration as the first symptom of COVID‐19 infection occurred, whereas other authors even described taste alteration as the only one symptom detected of this infection (Hjelmesæth & Skaare, 2020; Jang et al., 2020; Villalba et al., 2020).

A result was the identification of a statistically significant association between taste alteration, female gender and early age (Bénézit et al., 2020; Gelardi et al., 2020; Giacomelli et al., 2020; Jang et al., 2020; Lechien et al., 2020); however, one study not reported a significant association with early age (Yan et al., 2020).

Beltrán‐Corbellini et al. (2020) compared a cohort of 79 patients diagnosed with COVID‐19 to a cohort of 40 patients suffering from seasonal flu (as control) with similar demographic characteristics. Loss of smell and taste was found statistically significant in the cohort of COVID‐19 patients, 11 of them also reported dysgeusia and anosmia as the first symptom occurred.

Roland et al. (2020) reported a high specificity (73%) of dysgeusia and anosmia in diagnosing COVID‐19 and (Bénézit et al., 2020) found a 95% specificity of dysgeusia according to Wee et al. (2020) (98.7%, 95% CI 97.6–99.4%), but lower sensitivity (22.7%, 95% CI 16.4–30.2%).

Some authors considered dysgeusia and anosmia, regardless of the differences (Beltrán‐Corbellini et al., 2020; Lee, Min, Lee, & Kim, 2020; Roland et al., 2020; Vaira, Deiana, Fois, et al., 2020), furthermore, in some works (Lechien et al., 2020), a positive association between the two symptoms was reported (*p* < .001).

### 3.2 Oral blister and ulcers

At the present, few studies cited oral lesions during COVID‐19 infection; in detail, only one work by Martín Carreras‐Presas et al. (2020) reported three cases of diffuse oral pain, desquamative gingivitis, ulcers and blisters. However, only one of these was actually diagnosed with the COVID‐19: the patient was a female of 65 years old, who had blisters in her internal lip mucosa as well as desquamative gingivitis.

The authors recognized the case as Erythema Multiforme‐like eruption.

From literature analysis, oral manifestations were also reported associated with other dermatological alteration; Jimenez‐Cauhe et al. (2020) mentioned three COVID‐19 positive females, aged between 58 and 77 years old, with palatal macules and petechiae associated with Erythema Multiforme‐like eruption. This manifestation was found to occur on average 19.5 days after the presumed infection. In addition, Hedou et al. (2020) reported 1 case of herpetic stomatitis of 100 intubated patients.

### 3.3 Kawasaki‐like disease

One of the issues of interest during the COVID outbreak was found to be the potential association between Kawasaki disease (KD) and the Coronavirus infection.

The KD can display changes of the lips and oral cavity, including erythema, dryness, fissuring, peeling, cracking, bleeding of lips, “strawberry tongue.”

A deep investigation of this pathology could be useful for oral professionals in order to disclose potential differential diagnoses or perform early diagnosis. When KD occurs in association with COVID‐19, its clinical manifestations are worse when compared with clinical features reported in the literature. Therefore, in these cases, it was reported as Kawasaki‐like disease (Verdoni et al., 2020).

An Italian observational study by Verdoni et al. (2020) showed that, during the COVID‐19 outbreak, KD had a monthly incidence at least 30 times higher than the monthly incidence of the previous 5 years in the Bergamo district. The study reported 10 pediatric patients affected by this condition, 5 of them showed the classic form and 5 the incomplete form. Eighty percent of patients diagnosed with classic form presented changes of the lips or oral cavity, or both and one of them showed later cervical lymphadenopathy. Non‐exudative conjunctivitis associated with changes in the lips and oral mucosa was highlighted in one of the patients affected by Kawasaki incomplete form.

Furthermore, a case report from the United States described a 5‐year‐old patient, diagnosed with incomplete Kawasaki associated with fever (up to 39.4°C for 8 days), dry, cracked and erythematous lips, non‐exudative conjunctivitis and bilateral cervical lymphadenopathy without skin rash (Rivera‐Figueroa et al., 2020).

## 4 DISCUSSION

A wide spectrum of signs and symptoms were reported in association with novel COVID‐19, however, few studies highlighted oral clinical manifestations observed in patients diagnosed with this disease.

One of the most described oral symptoms was the taste alteration; indeed, a recent review of literature noted the 43.93% (95% CI, 20.46–68.95%) prevalence of gustatory dysfunction (Tong, Wong, Zhu, Fastenberg, & Tham, 2020). In this work, prevalence was reported ranged between 5.6% (Mao et al., 2020) and 92.64% (Bénézit et al., 2020), and in some case taste alteration was the first symptom of COVID‐19 (Gelardi et al., 2020; Hjelmesæth & Skaare, 2020; Melley et al., 2020; Villalba et al., 2020; Vinayachandran & Balasubramanian, 2020); no less, a statistically significant association was found with young age and female gender (Beltrán‐Corbellini et al., 2020; Giacomelli et al., 2020; Lechien et al., 2020; Lee, Huo, & Huang, 2020).

However, these results deserve to be more carefully evaluated due to some critical issues, considering that this is a very subjective symptom and it is difficult to diagnose. Primarily, it should be considered that several studies documented symptoms based on self‐reported and it could lead to an underestimation of them. Among all studies selected in our review, only the work by Lechien et al. (2020) assessed taste alteration using a validated questionnaire, the taste and smell disorders component of the National Health and Nutrition Examination Survey (NHANES). However, Vaira, Salzano, Deiana, and De Riu (2020) tested these alterations by means of different solutions (sweet, salted, acid and sour solutions) and they scored results. These three studies were found to be similar in terms of incidence of taste alteration, indicating values of 88.8, 73.6 and 51.5% respectively. Other studies that assessed this symptom based on self‐reporting resulted in a lower percentage of incidence (Wee et al., 2020).

In detail, Vaira supported this finding with three different studies assessing the taste alteration of COVID‐19 patients. In the first (Vaira, Salzano, Fois, Piombino, & De Riu, 2020), the symptom was tested using self‐reporting and the incidence was found to be of 19.4%, in the others (Vaira, Salzano, Petrocelli, et al., 2020) a test was used and the incidence was reported to be 73.6 and 51.5%.

With regard to the association between age and reported taste alteration, clarification is a duty. It is well known how altered taste perception is related with age and, in details, this association could be due to a decrease in saliva, drugs assumption, diabetes, malnutrition, neurological and oral mucosa disorders (Doty, 2018), all affections often associated with advanced age. This could lead to believe that the elders did not recognize the symptom during COVID‐19 infection.

Furthermore, the resulted correlation between female gender and taste alteration could be explained by the gender difference in terms of inflammatory reactions (Lechien et al., 2020), highly expressed in females or, more simply, by the physiological influence of hormones in taste perception (Di Fede, Majorana, Manfredi, Pentenero, & Giuliani, 2014).

Several authors of studies selected reported positive associations between COVID‐19 infection and smell and taste disorders (Bénézit et al., 2020; Lechien et al., 2020). However, some of them did not differentiate between the two symptoms (Beltrán‐Corbellini et al., 2020; Lee, Min, et al., 2020; Roland et al., 2020), therefore data about taste could be underestimated (Tong et al., 2020). Most authors indicate the presence of these symptoms in absence of rhinitis or nasal obstruction (Tong et al., 2020; Vaira, Salzano, Petrocelli, et al., 2020), leading to hypothesize a direct action of the virus in determining this symptom.

According to some scholars, a possible cause of taste alteration could be the presence in oral mucosa of ACE2 receptors, identified as the cellular receptors for SARS CoV‐2.

In detail, a work by (Xu et al., 2020) noticed the higher expression of ACE2 on tongue compared to other sites of oral mucosa. This could explain the findings reported and gives the reason for the independence of this particular symptom from smell alterations. Furthermore, ACE2 expression in epithelial cell of salivary glands, highlighted by (Xu, Li, Gan, Du, & Yao, 2020), is even higher than in the lung cells, indicating that salivary glands might be a potential target for COVID‐19. The glands and their ducts may be further damaged and therefore a healing process of healing acted by fibroblasts with a fibrous connective tissue formation may follow. This mechanism, according to Wang et al. (2020) could be the basis of early manifestations of COVID‐19 such as acute sialadenitis accompanied by pain and tumor in parotid and submandibular glands. This process could be followed by chronic sialadenitis due to a decrease in the secretion of saliva responsible for potential infections and stones in salivary ducts.

These changes in secretion of saliva deserve to be taken into account during the early diagnosis process, they could be responsible for taste alteration and oral complication due to hyposcialia (e.g., oral candidiasis, burning mouth [Campisi et al., 2008]).

With this in mind, the presence of hyposalivation or other salivary alterations needs to be carefully evaluated (Vinayachandran & Balasubramanian, 2020).

Lesions reported in the oral cavity are an important issue to assess and, where possible, to better frame: concerning them the knowledge is scarce. At the present, only Martín Carreras‐Presas et al. (2020) described in detail oral lesions during COVID‐19 infection, whereas other findings were reported by dermatological scholars describing oral manifestations of dermatological diseases (Jimenez‐Cauhe et al., 2020; Rivera‐Figueroa et al., 2020; Verdoni et al., 2020). In addition, in several studies reporting cutaneous changes, information about the location of lesions was not provided (Tang et al., 2020).

As reported by Martín Carreras‐Presas et al. (2020) and Hedou et al. (2020), elementary lesions (ulcers or vesciculo‐bullous lesions) can occur as in other viral infections. It is largely demonstrated that high levels of fatigue and stress could be associated with an increased risk of reactivation of the HSV (Forbes et al., 2019), a common condition in cases of flu.

Likewise, Erythema multiforme was largely reported in the literature in association with viral infections, for example, Adenoviruses, Enteroviruses, Herpes Viruses, with an occurrence of oral manifestations in 70% of cases, especially represented by blisters and ulcers located on lips or non‐keratinized mucosa (Scully & Bagan, 2008).

Considering these findings, it is possible that this virus provokes exanthematous lesions as other viral processes that we are used to diagnose in the dental practice.

Same considerations are viable for Kawasaki‐like disease. Indeed, one of the most reported risk factors of this disease is viral infection, such as Adenoviruses, Enteroviruses, Influenza A, Para‐influenza type 3; in detail, a study by Chang et al. (2014) highlighted a statistically significant association between Coronaviridae and KD, demonstrating also that the 60% of patients positive for Kawasaki had a virus infection affecting the respiratory tract.

To point out that Erythema Multiforme and KD pathogeneses could be linked to an inflammatory disorder affecting blood vessels, therefore we could assume that the oral manifestations of COVID‐19 can be traced back to ACE2 endothelial receptors.

A critical point of this narrative review has to be recognized in a few reported studies from Asian countries (China, Korea and Singapore) about this issue. As some authors yet reported, we have to take into account that different lifestyles might affect ACE2 expression (such as diet, cigarette smoking) causing different patterns of clinical manifestation among different countries with different lifestyles (Li, Zhou, Yang, & You, 2020).

There are some limitations to this review, due to the design of the studies included, that were case reports/series, or cross‐sectional or retrospective studies, therefore their scientific contribution could seem limited. This is an issue considered by authors, however, the current socio‐economic and medical matters required to deeply investigate and discover as much as possible about this severe infection: with this in mind, also weak studies were included in the review.

To conclude, we observed that oral signs and symptoms of COVID‐19 are not frequently recognized and reported by patients, together with the fact that they could be non‐specific of this disease. In this context, differential diagnosis with other chronic conditions is advisable, always deeply defining a correct and complete anamnesis of the patient.

However, several diseases present oral involvement as a first and only manifestation (Capocasale, Perno, Nocini, Albanese, & Zotti, 2020; Dalessandri et al., 2019; Dioguardi et al., 2016; Dioguardi et al., 2019; Porter, Mercadante, & Fedele, 2017): this issue has to be evaluated and therefore assessed also for this new infection.

In this regard, it could be actually useful that oral professionals evaluate and categorize all oral involvements of Coronavirus infection: this important action would allow to not underestimate and misdiagnose oral sign and symptom. With these findings in mind, we suggest that all specialists involved in oral health, especially dentists and dermatologists, could perform the oral examination in patients suspected or affected by SARS‐CoV‐2.

Furthermore, it is important to report the presence of any oral manifestations, especially until little is known. Indeed, the knowledge of oral signs and symptoms could be important in the dental setting for performing a more accurate triage of patients and for early diagnosis. For example, dental practitioners should include “recent onset of taste disturbance” or “oral ulcers” as questions of routinely anamnestic questionnaire and filtering out those who are at higher risk from attending primary healthcare environment.

The oral professionals must be conscious of COVID‐19 (Bizzoca, Campisi, & Muzio, 2020) and their valuable role in controlling outbreaks. Health care professionals, indeed, could apply up‐date triage before dental procedures and clinically identify early oral signs and symptoms of COVID‐19, they also refer the patients for further investigation to speed‐up self‐isolation procedures.

## CONFLICT OF INTEREST

The authors declare no conflict of interest.

## AUTHOR CONTRIBUTIONS

Giorgia Capocasale wrote the manuscript; Giorgia Capocasale and Riccardo Nocini conducted the conceptualization, the methology, the investigation and the formal analysis; Dario Donadello and Paolo Faccioni conducted data curation; Francesca Zotti provided review and editing; Massimo Albanese, Francesca Zotti and Dario Bertossi provided the validation; Francesca Zotti and Massimo Albanese reviewed the final manuscript.

## Data Availability

Data sharing is not applicable to this article as no new data were created or analyzed in this study.
